# Metformin vs Insulin in the Management of Gestational Diabetes: A Meta-Analysis

**DOI:** 10.1371/journal.pone.0064585

**Published:** 2013-05-27

**Authors:** Juan Gui, Qing Liu, Ling Feng

**Affiliations:** Department of Obstetrics and Gynecology, Tongji Hospital, Tongji Medical College, Huazhong University of Science and Technology, Wuhan, Hubei, China; Sapienza, University, Italy

## Abstract

**Background:**

Nowadays, there have been increasing studies comparing metformin with insulin. But the use of metformin in pregnant women is still controversial, therefore, we aim to examine the efficiency and safety of metformin by conducting a meta-analysis of randomized controlled trials (RCTs) comparing the effects of metformin with insulin on glycemic control, maternal and neonatal outcomes in gestational diabetes mellitus (GDM).

**Methods:**

We used the key words “gestational diabetes” in combination with “metformin” and searched the databases including Pubmed, the Cochrane Library, Web of knowledge, and Clinical Trial Registries. A random-effects model was used to compute the summary risk estimates.

**Results:**

Meta-analysis of 5 RCTs involving 1270 participants detected that average weight gains after enrollment were much lower in the metformin group (n = 1006, *P* = 0.003, SMD = −0.47, 95%CI [−0.77 to −0.16]); average gestational ages at delivery were significantly lower in the metformin group (n = 1270, *P* = 0.02, SMD = −0.14, 95%CI [−0.25 to −0.03]); incidence of preterm birth was significantly more in metformin group (n = 1110, *P* = 0.01, OR = 1.74, 95%CI [1.13 to 2.68]); the incidence of pregnancy induced hypertension was significantly less in the metformin group (n = 1110, *P* = 0.02, OR = 0.52, 95%CI [0.30 to 0.90]). The fasting blood sugar levels of OGTT were significantly lower in the metformin only group than in the supplemental insulin group (n = 478, *P* = 0.0006, SMD = −0.83, 95%CI [−1.31 to −0.36]).

**Conclusions:**

Metformin is comparable with insulin in glycemic control and neonatal outcomes. It might be more suitable for women with mild GDM. This meta-analysis also provides some significant benefits and risks of the use of metformin in GDM and help to inform further development of management guidelines.

## Introduction

In recent years, the morbidity of gestational diabetes mellitus (GDM) is increasing. Approximately 1–14% of all pregnancies are complicated by GDM, depending on the population studied and the diagnostic tests employed [1]. It has been defined as any degree of glucose intolerance with onset or first recognition during pregnancy [1]. GDM, the most frequent medical complication of pregnancy, is associated with several adverse outcomes over the short- and long-term for both mother and offspring [2]. First, the presence of GDM always accompanies an increased maternal risk for preeclampsia, cesarean section, and with an increased risk for developing type 2 diabetes (T2D) after pregnancy [3], [4]. In addition, there is an increased risk for neonatal death, still birth and congenital defects [5] resulting from excessive mother-to-fetus glucose transfer [6], [7]. Another major complication is macrosomia, which is a risk factor for instrumental delivery, cesarean section and shoulder dystocia during delivery and neonatal hypoglycemia directly after birth [8]. Furthermore, the influence of the intrauterine hyperglycemia environment might go with the children in later life [3], [9]. So the management of GDM is primarily aimed at controlling glycemic level to reduce the incidence of adverse pregnancy outcomes. The previous study has demonstrated that intensive treatment in women with GDM reduced birth weight and incidence of macrosomia in infants born to mothers who had participated in the intervention compared with women who had received routine care [10]. Therefore, active treatments - such as dietary therapy, exercise, oral hypoglycemic agents, insulin - are necessary to reduce the complications [11].

When an appropriate diet, alone or associated with physical exercise, does not suffice to control blood glucose levels in pregnant women, subcutaneous insulin therapy has been considered the standard for management of GDM [12]-[14]. However, insulin has several disadvantages including multiple daily injections, the risk of hypoglycemia and maternal weight gain [15]. It requires modification based on the patient's body mass index, glucose levels and lifestyle [16]. Therefore, detailed guidance for dose change of insulin is necessary to ensure the safe self-administration of insulin. Meanwhile, substantial costs of health education on the safe use of insulin as well as the cost of the drug itself are followed. Naturally, safe and effective oral therapy would be more acceptable even highly desired for women with GDM [2], [17]. However, it is essential to comprehend the effects of oral hypoglycemic agents on both maternal and neonatal outcomes for the women with GDM. Metformin, as the first line medication for T2D, sits in the candidate list. Given that metformin has been found to have a maternal-to-fetal transfer rate of 10–16% [18], [19] which might be associated with fetal anomalies, potential adverse effects for mothers and the newborns after delivery, it has not been widely used in GDM. Nowadays, increasing studies focus on examining the efficiency and safety of metformin in the management of GDM. However, some are case-control trials [20]-[22], some are observational studies [23], others are randomized controlled trials (RCTs) but with small samples lacking the power to draw confirmative conclusions on the relative risks and benefits of metformin for GDM. So the use of metformin is still controversial in pregnant women, therefore, the aim of this meta-analysis is to provide pooled estimates of RCTs comparing the effects of metformin with insulin on glycemic control, maternal and neonatal outcomes in GDM.

## Methods

### Search Strategy

We searched the databases including Pubmed, the Cochrane Library, Web of knowledge, and Clinical Trial Registries (Last search was updated on November, 2012). We used the key words “gestational diabetes” in combination with “metformin”, and examined the reference lists of the obtained articles, reviews excluded. When necessary, we contacted authors of original studies for additional data.

### Study Selection

Studies were included if they met the following criteria: participants were patients with GDM; the study design was a randomized controlled trial; it compared metformin with insulin; the study measured glycemic control, one or more maternal and neonatal outcomes. Retrospective studies, observational studies, case series, and studies with a crossover design were excluded.

### Data Extraction and Quality Assessment

We extracted following information from the eligible studies: author name, publication year, country, sample size, method of randomization, allocation concealment, blinding, dose of interventions, maternal and neonatal outcomes and so on. Maternal outcomes contain glycemic control, incidence of cesarean section, weight gain after enrollment, pregnancy induced hypertension (PIH), preeclampsia (blood pressure >140/90 mmHg with proteinuria >0.3 g/24 h), preterm delivery (<37 weeks of gestation), gestational age at delivery, shoulder dystoscia, etc. Neonatal outcomes include hypoglycemia, birthweight, neonatal intensive care unit (NICU) admissions, large for gestational age-birth weight >90^th^ percentile (LGA), small for gestational age-birth weight <10^th^ percentile (SGA), respiration distress syndrome (RDS), hyperbilirubinemia (>7 mg/dl), etc. Two reviewers (Gui and Liu) independently performed the literature search, study selection and data extraction. Differences in opinion were resolved by consensus with a third reviewer (Feng).

### Statistical Analysis

All analyses were performed using Review Manager 5.1 (Nordic Cochrane Centre). The heterogeneity was evaluated statistically by the Chi-squared test (*P*<0.1) and graphically using a forest plot analysis. A random effects model, which considered both within- and between- study variation, was used for all the meta-analysis. For continuous outcomes we calculated standard mean differences (SMD) and 95% confidence intervals (CI). For dichotomous outcomes we calculated odds ratio (OR) and 95% CI.

## Results

### Search Results

**Figure 1:**
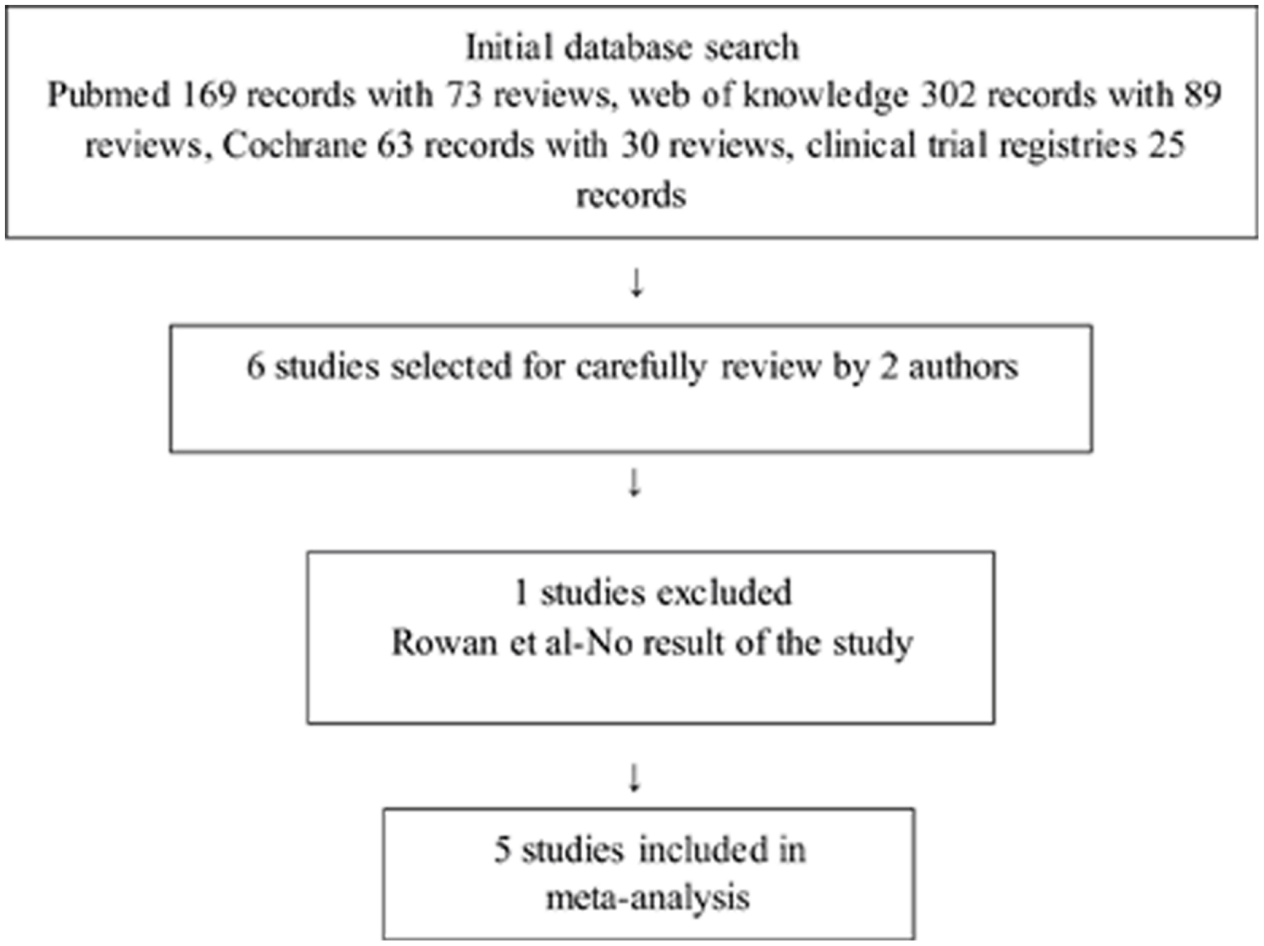
Flow of study identification, inclusion, and exclusion.

Figure 1 shows the study selection process. The search strategy used in this review resulted in identification of 169 records with 73 reviews in Pubmed, 302 records with 89 reviews in web of knowledge, 63 records with 30 reviews in Cochrane library, and 25 records in Clinical Trial Registries. After rejecting the reviews and screening the titles and abstracts, 6 trials involving GDM and metformin were assessed for eligibility. Of the 6 studies, 5 studies comprising 1270 participants were included in the meta-analysis. The trial excluded was: Rowan [24]-it was a trial in process with no maternal or neonatal outcomes.

### Study Characteristics

Table 1 shows the characteristics of the included studies. Table 2 shows the criteria used for diagnosis and criteria for starting medical treatment. The quality assessments of the included studies Tertti [25], Alavi [26], Ijas [17], Rowan [27], Moore [28] are presented in Table 3.

**Table 1:** Characteristics of included studies.

Author	Country	Patients on	Patients on	Metformin group	Dose of	Dose of	Side effects of	Loss to
		metformin	insulin	requiring insulin	insulin (u)	metformin (mg)	metformin	follow–up
Moore et al (2007)	USA	32	31	0 (0%)	Not reported	1000–2000	0	0
Rowan et al (2008)	Australia,	363	370	168 (46.3%)	30–90 (50)	1750–2500	39	0
	New Zealand							
Ijas et al (2010)	Finland	47	50	15 (31.9%)	30	750–2250	5	0
Alavi et al (2012)	Iran	80	80	11 (14%)	Not reported	1000–2500	6	8
Tertti et al (2012)	Finland	110	107	23 (20.9%)	2–42	500–2000	2	4
Patients (n)		632	638					

**Table 2:** Criteria for diagnosis and starting medical treatment of GDM.

Author	Criteria for diagnosis of GDM					Criteria for starting medical treatment	
	Loading	Fasting,	1 h, mg/dl	2 h, mg/dl	3 h, mg/dl	Fasting, mg/dl	Postprandial, mg/dl
Moore et al (2007)	100 g	105	190	165	145	105	120
Rowan et al (2008)	75 g	99		126		97.2	120.6
Ijas et al (2010)	75 g	95.4	198	172.8		95.4	120.6
Alavi et al (2012)	100 g	95		120		95	120
Tertti et al (2012)	75 g	95.4	180	154.8		99	140.4 (1 h)

**Table 3:** Quality assessment of included studies.

Study	Randomization	Concealment	Selection	Group	Assessors	Outcomes
			criteria	comparability	blinding	intention to treat
Moore et al (2007)	Yes	Doubtful	Inclusion yes	BMI more in	No	Yes
			Exclusion no	Metformin		
Rowan et al (2008)	Yes	Open-label	Inclusion yes	Yes	No	Yes
			Exclusion yes			
Ijas et al (2010)	Yes	Open-label	Inclusion yes	Yes	No	Yes
			Exclusion yes			
Alavi et al (2012)	Yes	Single-blind	Inclusion yes	Yes	No	Yes
			Exclusion yes			
Tertti et al (2012)	Yes	Open-label	Inclusion yes	Yes	No	Yes
			Exclusion yes			

### Main Maternal Outcomes

#### Glycemic control

Average fasting and postprandial glycemic levels were reported in 3 studies. They were slightly lower in the metformin group as compared with the insulin group, but the difference was not statistically significant in fasting glycemic control (*P*_heterogeneity_ = 0.09, n = 956, *P* = 0.92, SMD = −0.01, 95%CI [−0.28 to 0.25]) and in postprandial glycemic control (*P*_heterogeneity_ = 0.19, n = 956, *P* = 0.2, SMD = −0.14, 95%CI [−0.35 to 0.07]).

When compared with insulin group, average postprandial glycemic levels at the first week after randomization were significantly lower in the metformin group (*P*_heterogeneity_ = 0.91, 2 trials, n = 893, *P* = 0.002, SMD = −0.21, 95%CI [−0.34 to −0.07]). There was no significant difference between the two groups in average HbA1c% levels at gestational 36–37 week (*P*_heterogeneity_ = 0.89, 2 studies, n = 356, *P* = 0.88, SMD = −0.02, 95%CI [−0.22 to 0.19]) and in average fasting glycemic levels at the first week after randomization (*P*_heterogeneity_ = 0.24, 2 studies, n = 893, *P* = 0.59, SMD = 0.05, 95%CI [−0.13 to 0.23]).

#### Main maternal risks

**Figure 2:**
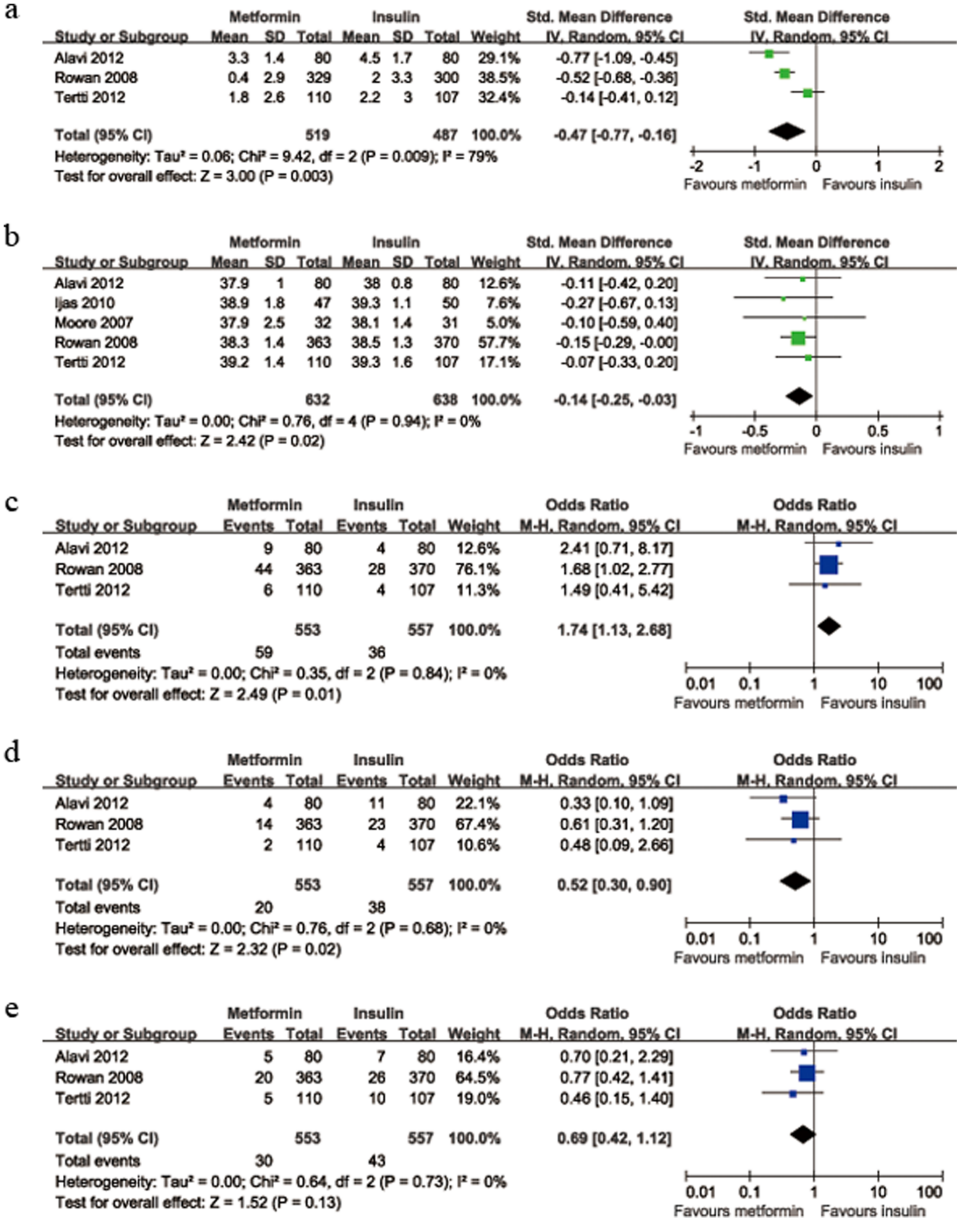
Forest plot of main maternal risks comparing metformin with insulin. a: weight gain after enrollment; b: gestational age at delivery; c: incidence of preterm birth; d: incidence of PIH; e: incidence of preeclampsia. SMD: standard mean differences; CI: confidence intervals; OR: odds ratio; PIH: pregnancy induced hypertension.

The data on average weight gain after enrollment were available from 3 trials. There was significant heterogeneity between these trials (*P*_heterogeneity_ = 0.009). When compared with insulin group, average weight gains after enrollment were much lower in the metformin group (n = 1006, *P* = 0.003, SMD = −0.47, 95%CI [−0.77 to −0.16]); average gestational ages at delivery were significantly lower in the metformin group (*P*_heterogeneity_ = 0.94, 5 trials, n = 1270, *P* = 0.02, SMD = −0.14, 95%CI [−0.25 to −0.03]); preterm birth rate was significantly more in metformin group (*P*_heterogeneity_ = 0.84, 3 studies, n = 1110, *P* = 0.01, OR = 1.74, 95%CI [1.13 to 2.68]); PIH rate was significantly less in the metformin group (*P*_heterogeneity_ = 0.68, 3 trials, n = 1110, *P* = 0.02, OR = 0.52, 95%CI [0.30 to 0.90]). There was no significant difference in the preeclampsia rate between the two groups (*P*_heterogeneity_ = 0.73, 3 studies, n = 1110, *P* = 0.13, OR = 0.69, 95%CI [0.42 to 1.12]). Figure 2 shows the details of main maternal risks.

### Main Neonatal Outcomes

**Figure 3:**
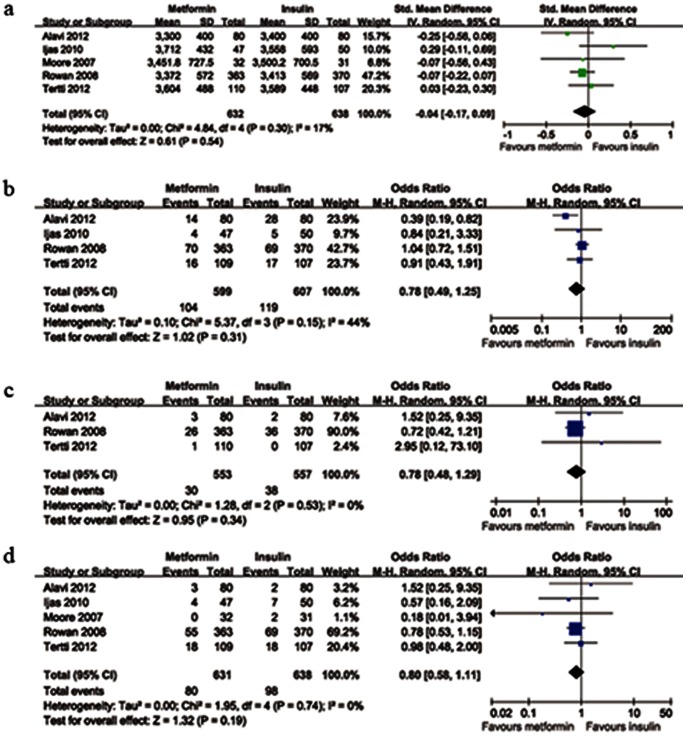
Forest plot of main neonatal risks comparing metformin with insulin. a: birth weight; b: incidence of LGA infants; c: incidence of SGA infants; d: incidence of hypoglycemia. SMD: standard mean differences; CI: confidence intervals; OR: odds ratio; LGA: large for gestational age; SGA: small for gestational age.

Average birth weights were reported in 5 studies. There was no significant heterogeneity between these studies (*P*_heterogeneity_ = 0.30). Average birth weights were slightly lower in the metformin group as compared with the insulin group, but the difference was not statistically significant (n = 1270, *P* = 0.54, SMD = −0.04, 95%CI [−0.17 to 0.09]). When compared with insulin group, the pooled result showed no significant difference between the metformin and insulin groups in LGA infants rate (*P*_heterogeneity_ = 0.15, 4 trials, n = 1206, *P* = 0.31, OR = 0.78, 95%CI [0.49 to 1.25]); in the SGA infants rate (*P*_heterogeneity_ = 0.53, 3 studies, n = 1110, *P* = 0.34, OR = 0.78, 95%CI [0.48 to 1.29]); in hypoglycemia rate (*P*_heterogeneity_ = 0.74, 5 trials, n = 1269, *P* = 0.19, OR = 0.80, 95%CI [0.58 to 1.11]). Figure 3 shows the details of main neonatal risks.

### Others

There was no significant difference between the two groups in the cesarean delivery rate (*P*_heterogeneity_ = 0.25, 5 studies, n = 1270, *P* = 0.75, OR = 0.95, 95%CI [0.68 to 1.32]) and in the incidence of shoulder dystocia (*P*_heterogeneity_ = 0.79, 4 studies, n = 1173, *P* = 0.18, OR = 0.58, 95%CI [0.26 to 1.29]).

There was no significant difference between the two treatment groups in the incidence of NICU admission (*P*_heterogeneity_ = 0.62; 5 trials, n = 1269, *P* = 0.22, OR = 0.84, 95%CI [0.63 to 1.11]); in the incidence of RDS (*P*_heterogeneity_ = 0.67; 4 trials, n = 1173, *P* = 0.34, OR = 1.52, 95%CI [0.64 to 3.59]); in the incidence of hyperbilirubinemia (*P*_heterogeneity_ = 0.13; 3 trials, n = 320, *P* = 0.95, OR = 0.98, 95%CI [0.44 to 2.17]); in birth defect rate (*P*_heterogeneity_ = 0.39; 3 trials, n = 1110, *P* = 0.56, OR = 0.83, 95%CI [0.45 to 1.55]); in birth injury rate (*P*_heterogeneity_ = 0.31; 2 trials, n = 950, *P* = 0.71, OR = 0.86, 95%CI [0.40 to 1.87]); in phototherapy rate (*P*_heterogeneity_ = 0.57; 3 trials, n = 1109, *P* = 0.98, OR = 1.00, 95%CI [0.65 to 1.56]); in the PH of umbilical-cord artery (*P*_heterogeneity_ = 0.87; 4 trials, n = 665, *P* = 0.59, SMD = 0.04, 95%CI [−0.11 to 0.19]); in the 5-min Apgar score (*P*_heterogeneity_ = 0.39; 3 trials, n = 376, *P* = 0.31, SMD = −0.11, 95%CI [−0.31 to 0.10]).

After eliminating some data of the participants supplementing insulin from metformin group, the incidence of preterm birth was still significantly higher in metformin group than in insulin group (*P*_heterogeneity_ = 0.85, 3 trials, n = 942, *P* = 0.02, OR = 1.77, 95%CI [1.09 to 2.87]). There was still no significant difference between the two groups in birth weight (*P*_heterogeneity_ = 0.66, 5 trials, n = 1255, *P* = 0.25, SMD = −0.06, 95%CI [−0.18 to 0.05]); in the incidence of LGA infants (*P*_heterogeneity_ = 0.1, 4 trials, n = 1191, *P* = 0.25, OR = 0.72, 95%CI [0.42 to 1.25]); in the incidence of hypoglycemia (*P*_heterogeneity_ = 0.73, 5 trials, n = 1101, *P* = 0.19, OR = 0.78, 95%CI [0.54 to 1.13]). The fasting blood sugar levels of OGTT were significantly lower in the metformin only group than in the supplemental insulin group (*P*_heterogeneity_ = 0.03, 3 trials, n = 478, *P* = 0.0006, SMD = −0.83, 95%CI [−1.31 to −0.36]).

### Adverse Events

Rowan [27] reported one fetal death in the insulin group. Moore [28] reported one intrauterine fetal death because of acute asphyxia probably induced by a cord accident in the metformin group.

## Discussion

In the meta-analysis, 3 studies measured fasting and postprandial blood sugar and 2 detected the HbA1c% to check the efficiency of metformin. The results are the same as the previous reviews [14], [29] that metformin is comparable with insulin in glycemic control. Metformin reduces hyperglycemia by suppressing hepatic glucose output (hepatic gluconeogenesis), increasing insulin sensitivity and enhancing peripheral glucose uptake [30]. These effects are potentially useful during pregnancy when glucose control deteriorates with changes to insulin resistance [16]. In addition, we found that the average postprandial glycemic levels at first week after randomization were significantly lower in the metformin group. This finding indicates that metformin group reached glucose targets sooner. The reason might be that it takes time for the participants to master the usage and dose-computation of insulin.

Moreover, our findings, in accordance with the results of previous reviews [14], [29], suggest that neonatal outcomes don't deteriorate with the use of metformin as compared with insulin in short term. At the same time, the results of studies for the long-term impact of metformin use are encouraging. A study followed the neonates whose mothers received metformin and found that they displayed normal weight and social and motor skills at 6 months and there were no differences in height, weight, motor, or social skills between the neonatal groups at 18 months [31]. Moreover, the results of Rowan et al. [32] on this issue are both encouraging and reassuring which intrigue the possibility of benefit in children and adolescents with intrauterine exposure to metformin.

Besides, we found some significant benefits and risks of metformin seldom demonstrated before in the maternal outcomes. One benefit concerns maternal weight gain in pregnancy. Metformin inhibits hepatic gluconeogenesis and glucose absorption and stimulates glucose uptake in peripheral tissues, with the effect of reducing weight gain [33]. Obesity is always the high risk for metabolic diseases, so less weight gain, less incidence of other complications. Another is the lower morbidity of PIH. The possible explanation might be that metformin has complex properties on endothelial functions and reactive oxygen species production [34], so as to reduce the endothelial activation and maternal inflammatory response of insulin resistance. On the other hand, we found that average gestational ages at delivery were significantly lower in the metformin group, and the incidence of preterm birth was significantly more in metformin group as compared with insulin group even after eliminating the data of supplemental insulin group from Rowan’s [27] study. This implicates that metformin might have an unrecognized effect on the labor process and informs that the use of metformin in pregnancy should be deliberate.

In this meta-analysis, the incidence of requiring additional insulin to achieve euglycemia was especially high in the study of Rowan [27] (46.3%). Various racial groups and glycemic targets might contribute to the differences among studies.We found that women requiring supplemental insulin had higher fasting glycemic concentrations in OGTT. This indicates that metformin might be especially suitable for mild GDM patients and provides information for the further development of GDM management.

Heterogeneity in the outcomes of average fasting glycemic control and weight gain after enrollment might result from the various ethnic groups, study designs and entry criteria. The different gestational ages at enrollment might also result in heterogeneity in weight gain. Before we come into definite conclusions, several limitations should be considered in this meta-analysis. First, only a few studies fulfilled the inclusion criteria. Second, some important outcomes were not reported in every included study. Third, most of the studies included were open-label RCTs with high risk of performance and detection bias. Last but not least, the hypotheses for the studies were not the same. Rowan [27] used a superiority design to assess whether insulin was superior to metformin, while Tertti [25] designed a non-inferiority study to evaluate whether there was a difference of effect between the two treatments. Funnel plots were not used for assessing the publication bias in the meta-analysis in that the small number of included original studies render unreliable indicators of publication bias.

In conclusion, metformin could be used in women with GDM in view of the comparative glycemic control and neonatal outcomes, especially for those mild GDM patients. However, the risk of preterm birth could not be ignored. Clinicians should weigh in practice according to the condition of the patients. Further studies with larger sample sizes must be completely designed to assess maternal and neonatal complications and to evaluate long-term follow-up of children for the safety of metformin as a universal treatment in GDM patients.

## Supporting Information

Checklist S1 **PRISMA Checklist.**
(DOC)
